# Parasitic Origin of Disease

**Published:** 1874-03

**Authors:** E. A. Parkes


*Parasitic Origin of Disease.* From Report on Hygiene, by E. A. PARKES, M.D., in British Army Medical Report for 1871, published April, 1873.

The origin of diseases from entozoa, or from low forms of life, fungoid or bacteroid, is so important a subject that it seems necessary to give a brief retrospect of what has been done this year. The progress of observations seem to make it clear that the true fungi must be separated from the class called schizomycetes by Nægeli and DeBary, which includes the bacteria and allied forms. The true fungi produce, for the most part, very obvious diseases, which are situated on the surface of the body or in cavities easily accessible to the air. The schizomycetes appear in all parts of the body, and have been found in the interior of living cells, and may be thus transported everywhere.

DISEASES IN MEN PRODUCED BY ENTOZOA.

*Filaria sanguinis hominis.*

The most remarkable discovery of the year is that of Dr. T. Lewis, Assistant Surgeon Army Medical Department in Calcutta. He had discovered, some years ago, in the urine of persons affected with chyluria, the young of a small worm belonging to the filaridæ, and he has now found that this worm exists in immense quantities in the blood, and emerges through the kidneys, and occasionally through other channels, as in one case by means of a discharge from the inner canthus of the eye.

The worms are easily detected in the blood, and exist there in great numbers without apparently causing any marked symptoms except those arising from their passage into the urine.

Another parasite has been also discovered by an army medical officer, Dr. Welch. A soldier from India died of phthisis five days after landing at Netley. In the jejunum, immediately beneath the mucous coat, was an oral prominence like a rice grain; this was found to be caused by a parasite .13– inch in length, and .053– inch in thickness. There was a chitinous investing membrane, and at one end a retractile proboscis with a number of hooklets; there was no mouth or intestines, and no distinct segmentation. This is the first time a representative of the acanthocephala has been discovered in the human body, though echinorhynchus are common in birds and fishes. In this man it produced no symptoms.

Dr. Wising, of Stockholm, describes a case of balantidium coli. A man became affected with diarrhea, and passed thin, pea-souplike stools. Under the microscope there were remains of food, lymphoid cells, blood, and large numbers of small white worms, which were found to be the balantidium coli described by Malmsten in 1856.

BILHARZIA HEMATOBIA.

Dr. Cattell, of the 10th Hussars, who served for five years in Natal, informs me that the endemic hematuria is almost confined to boys in the Dutch town of Maritzburg; girls do not suffer, and adults only slightly. The drinking water at Maritzburg is taken from open water-courses running along each street, which is often fouled by animals drinking, by ducks, and by surface washings. The boys play in this water, and no doubt drink it in the hot weather. During a residence there of five years, Dr. Cattell had no case among the soldiers' children. The drinking water for the troops is taken from a separate water-course, higher up than that of the town's supply. Dr. Cattell is impressed with the opinion that the drinking water is the source of introduction.

FUNGOID ORIGIN OF DISEASE.

No great advances have been made in this division of the subject. Tilbury Fox has described and figured* the spores and mycelium of the tricophyton tonsuraus in dust deposited from the air of schools in which ring-worm is prevailing. He figures also the epithelium, with apparently the spores of the fungus in or on it.

BACTEROID ORIGIN OF DISEASE.

Before noticing the diseases which have been attributed to bacteria, during the last year, it may be interesting to notice two papers by Oscar Grimm, of St. Petersburg,† and also to extract a few passages from a work by Dr. Eidam.

Grimm first discusses the significance of the terms vibrio, bacterium, spirillum, etc., and then gives a statement of the effect of reagents on vibriones. Strong sulphuric and hydrochloric acids dissolve vibriones; weak solutions act slowly; acetic acid dissolves them very slowly; chloride of ammonium kills them, and then dissolves them; creosote makes the organisms clearer, and the vacuole is then clearly seen; iodine colors them brown; carmine, red; alcohol and ether slowly dissolve them. All the tests show that they are formed of a substance like protoplasm; they are indifferent to the electric current. Grimm describes their movements and their still conditions very carefully. Like other observers, he was not at first able to see the clearage of vibrios, but he at last succeeded in doing so, in both vibrios and spirillum; and he also observed a very interesting phenomenon, viz., the conjugation, or union, of the one-jointed vibriones and their fusion into a two-jointed

* Lancet, January 6, 1872.

† Archif fur Mikroscop Anatomie, B. viii and ix.

link; and subsequently the union of other vibriones, so as to form a many-jointed rod. This union or copulation has been seen in the bacteria of the splenic apoplexy, and in the spirillum undula. He therefore remarks that the single joint is the individual and not the chain of rods. With respect to the origin of the splenic apoplexy, or carbuncular (milz-brand) bacteria, Grimm believes that they are developed out of the protoplasm of the white blood cells. He asks the question, are the germs of the vibriones then present in these blood corpuscles? If so, they are so minute as to be undiscoverable. He considers the spirillum volutans to be only the union of two samples of spirillum undula. So also bacterium enchelys can form long chains, and produce vibrio rugala, or vibrio bacillus. These two last named vibrios are distinguished only by the size of the joints. He has seen all intermediate forms between vibrio lineola and vibrio subsilis. In fact, he doubts whether any distinction can be drawn between the different kinds of vibrios, and he recognizes, indeed, only two forms, viz., spirillum and vibrio. He includes under the term vibrio, the genera vibrio and bacterium of Ehrenberg. He describes, however, a new form, which, if it is really a vibrio, would constitute a third genus.

He has satisfied himself that the vibriones need atmospheric air as nutriment, and also that they are nourished by the absorption of fluid. He has seen them attack spores and acquire a green color, while the contents of the spores were much lessened in amount. He has seen the vibrios take on in the same way a red color from a red fluid which they fed on; and when the nourishment was colorless, the vibrios were also without color.

With regard to the systematic place of the vibriones, he is inclined to put them by the side of the phycochromaceæ, which resemble the vibriones in structure, in their increase through simple division, in their movements, and lastly, in their formation of colonies, with separation of a gelatinous mass.

Some single oscillarineæ, however, resemble vibriones so much that they are distinguished with difficulty. Although there is this resemblance, there is no identity. He agrees with Cohn, that "the "phycochromaceæ may well have been the first organisms on the "earth, as they can only grow in hot, strongly saturated saline "solutions. I think that these organisms, whose joints may be "considered either plants or animals, divide into two branches, "from which the formation of the animal and vegetable characters "takes its rise."

The second author, Dr. Eidam, analyzes with great care the micrococcus theory of Hallier, and his doctrines of infectious diseases; and then passes in review the objections taken by DeBary and Hoffman to Hallier's assertions, and states also the views of Karsten and Bonorden on fungoid development. He sums up his own conclusions in terms as follows: Very important differences of opinion now exist among mycologists. Bonorden denies all alternation of generation among fungi; DeBary takes a middle course on this point; and Hallier makes the greatest possible use of metamorphoses, and his theory is indeed otherwise impossible. In no other natural science are the differences of opinion greater than in mycology. As regards the infectious diseases, it is extremely probable (ausser ordeutlich wahrscheinlich) that they are caused by fungi or similar bodies.

If it could be decidedly made out that the plasma of spores and mycelium disintegrated in order to form micrococci, and if these little granules could increase, and under favorable circumstances go on to new plants, the present cell theory would be greatly changed. The micrococcus theory has one support; the spores of peronospora, cystopus, etc., divide their contents into globular balls, which individualize themselves more and more, in order finally to take the form of swarms, which burst the spore-coats, and these swarms can bud and develop. If in these spores of large size there is such a division of plasma, and a further development of parts so arising, cannot something similar occur in other spores? The difficulties of experimenting are immense. In cultivating the products of disease, only fresh and bacteria-free substances must be used; blood taken (with all precautions) direct from the body is the best. Nevertheless, in almost every sample of healthy blood, there are many little corpuscles with dancing movements, which it is impossible to distinguish from micrococci. Eidam proceeds to point out the other great sources of fallacy in cultivation experiments, and finally concludes that the micrococcus theory has almost everything against it. It may indeed be questioned, he says, if, on account of their excessive minuteness, these objects can be dealt with with our present instruments, and if it is possible at present to close this controversy. Perhaps even our whole mode of investigation may be wrong, and renewed investigation is certainly indispensable.

It will be seen, then, that the impression made upon an observer of great authority is, that the question is at present impossible of solution, as far as the micrococcus theory is concerned, but on the whole he is adverse to this theory. At the same time he is impressed strongly with the opinion that the infectious diseases are of parasitic origin, and are caused by fungi or some similar growths. In reviewing the observations made during the last year, it will be seen how far fresh evidence has been brought; but the conclusion must still, I think, be that we must yet hesitate before considering that the parasitic theory of infectious diseases has been established.

SPECIFIC DISEASES SUPPOSED TO BE CAUSED BY BACTERIA.

The evidence which has been brought forward during the last year on this subject is interesting in a high degree. As regards cholera, the careful observations of Drs. Lewis and Cunningham, in Calcutta, seem to have disproved the possibility of either fungi or bacteria being the cause of cholera. In last year's report I gave the evidence of D. D. Cunningham in respect of the discharges, which agreed with that of Lewis on the same point, as showing no such plants as constant constituents. This year both observers have published a joint report on the blood in cholera. When the fresh blood was examined in 128 instances, with very high powers ($\frac{1}{25}$ of Powell, and $\frac{1}{12}$ immersion lens of Ross), no fungoid spores or bacteria, or anything which could be referred to either class, was found; and when the blood was kept, bacteria only appeared in a small number of cases. As far, then, as these fluids (the intestinal discharges and the blood) are concerned, it appears that these high powers failed to detect fungi or bacteria, and that it is in the highest degree improbable that either were present and undetected.

Very different is the case of some other diseases, if the observations of several observers, and especially of Professors Coze and Feltz, of Strasbourg, may be trusted. They assert that in the blood of septicæmia, typhoid fevers and puerperal fever, there is present a linked or chained bacterium, which they term (after Dujardin) *bacterium catenula*, and they believe that the growth of these bacteria is the efficient cause of these diseases; indeed they go further, and assert "that every infectious disease is of bactiferous nature," although the form of the bacteria is different in small-pox, scarlet fever and measles, from what it is in typhoid. The activity and specifity (to coin a word) arise, they conceive, not because they are the carriers of a special soluble principle, but from the rapidity of their multiplication; the form and size of the bacteria depend on the soil in which they are reproduced and multiplied. The various phenomena of the infectious diseases are supposed to be dependent on pathological localization, and these localizations arise from the rapid growth of bacteria; from the detritus formed from them when dead; from the leucocytes which are often simultaneously developed in excess; and from fibrillary deposits of fibrine. The authors attempt to prove these assertions (which, if correct, would largely modify our views of infectious diseases) by microscopic examination, by experiments, and by clinical observations. In their microscopic inquiries they employed Nachet and Verick's glasses (immersion and otherwise), and have worked up to 1,600 diameters, and they used distilled water, the steam of which had passed through a red-hot porcelain tube. The diseases treated of are pyæmia and septicæmia, typhoid fever, small-pox, scarlet fever, measles, and puerperal fever. They also give a chapter on the cultivation of infusoria and on the nature of bacteria. They distinguish between septicæmia and pyæmia, as is now usually done, though the two are often mixed. With respect to septicæmia, the authors showed in 1866 that the blood of animals infected with a poisonous liquid is itself infectious, and that the red globules of the blood are then altered, and a multitude of bacteria, etc., exist, and that in successive inoculations death ensues more and more rapidly, so that the infectious element seems to gain in activity as it passes through the same organisms. They now support these operations by numerous experiments on rabbits, which are, on the whole, strongly confirmatory of those made by Burdon-Sanderson, and presently to be recorded. Passing over the changes they describe in the red globules, in the white cells and in the fibrine (fibrillary deposits), and referring only to the infusoria, they found always single, double or multiple corpuscles, usually in chains, but looking like little worms. Sometimes they saw the whole element with its grayish or slightly yellowish color; sometimes merely a pale or blackish point (according to the illumination), which was owing to an element seen from above and presenting one end; these points measured .0016 mms.; the complete elements .004 to .02 mms. in length, and .066 mms. in thickness. They had little activity, and resembled bacteria rather than vibriones. They die in the body, and especially in the lungs. The cause of death in septicæmia is considered to be a profound alteration in the blood, tending to, but not usually reaching, putridity, and induced by the countless myriads of bacteridia. In pyæmia there is also often the presence of bacteria, but because there is usually coincident septicæmia, there is in addition or alone numerous embolisms arising from substances introduced mechanically into the circulation (pus, white globules, substances from inflamed veins), and these may exist without any bacteria. The authors thus draw a broad line between the two affections.

In typhoid fever, experiments were made with blood taken from human typhoid patients and injected into the blood of rabbits. An infectious disease (with implication of Peyer's patches) was given to the rabbits, whose blood was able to communicate the disease to other rabbits. The dried and powdered blood of an infected rabbit preserved its power to produce infection for more than a year. In the blood of men and rabbits there were (in addition to alterations in the red and also largely in the white globules), a great number of bacteria, smaller in size than in the septicæmic blood; they were often divided, as in septicæmia, into three, four or five segments (*B. catenula*). The evidence of the presence of bacteria in typhoid blood seems to be complete, but it is, of course, still an open question whether they are the cause of the typhoid fever, and it even appears from some of the *post mortem* histories that bacteria were not always found in the blood. The examinations of typhoid blood during life appear also to have been very few in number.

With regard to small-pox, the observations of Chauveau and Sanderson have proved that the infectious property resides in the solid glistening particles. Whether these glistening particles are bacteroid or not is a matter of dispute. Beale, their discoverer (in

1865), considers* that in vaccine lymph they consist "of a peculiar kind of living matter, the smallest particle of which, when supplied with its proper pabulum, will grow and multiply." Variolous pus contains similar particles, and in both cases they are portions of bioplastic matter. Three years afterward Keber described inconceivably minute organic corpuscles in variolous blood, in which a lively cellular growth took place, and Hallier and Zurn have observed similar particles in the lymph of the sheep-pox as well as in human small-pox. Dr. F. Cohn† has now convinced himself that these particles are "living uni-cellular organisms belonging to the group of the so-called globular or sphere bacteria (*Kugel bacterien*)." He considers that he has absolutely proved this, and he details his method (which is based on that of Burdon-Sanderson), by which be believes he has excluded all chance of foreign admixture. The appearances of vaccine and variolous lymph were identical; there were numerous extraordinarily small globular particles with no proper movement, but with only molecular movement; they were easily overlooked, as their refraction power is the same as that of serum. The immersion lens finds them most easily; their size is under .001 mm., perhaps about one-half or three-fourths of this size; there are some larger bodies which Cohn takes to be clearly cells. At first the molecules are separate, but they hang together like a figure of 8; these double cells increase after a short time, and there may be four or more hanging together and forming chains, and in one or two hours there are chains of eight divisions, and sarcina-like bodies are formed, and then all sorts of groups appear. These bodies Cohn considers to be the same as the micrococci of Hallier, but he does not desire to use this word, which may give rise to misconception, and proposes the name of "microsphœra," as identical with "Kugel-bacteria," and places them, of course, in the family of the schizomycetes. With respect to the question whether these "microsphœræ" are the bearers of the contagion, Cohn alludes to Chauveau's investigations, and considers it is most probable that the question must be answered in the affirmative. The authority of Ferdinand Cohn in a matter of this kind is so great that it would seem we must admit that the sinall points first described by Beale are really bacteria; at the same time, Beale has worked with such high powers, and is so expert in these investigations, that a further inquiry may perhaps still be demanded before Cohn's view is definitely admitted. Coze and Feltz state that they found articulated bacteria in immense quantities in the blood of the human subject attacked with small-pox, and, on injecting this blood into rabbits, they produced feverishness and bacteria similar to those in the variolous blood which were found in the rabbits' blood. In variolous blood

* "Disease Germs," 2d Edition.

† "Virchow's Archiv," Band 55, page 229. 1872.

they have also found round globules, armed with points, which they consider to be bacteria fixed to a globule. They also noticed many deposits of fibrine, as in typhoid fever. The kind of bacteria, they state, resembled the *B. bacillus* of Pasteur, and the *B. termo* of Muller, and are quite different in aspect from those of septicæmia and enteric fever, as in septicæmia the transmission of the virus through successive rabbits seemed to heighten the virulence of the poison.

In scarlet fever blood these authors have also found bacteria, which, when transported into the system of rabbits, largely increased in size. In man their length was .0006 mm., and their breadth .0002 mm.; in the rabbit they were eight times as big. The injection of human scarlatinal blood into rabbits produced a fatal, feverish disease.

In measles, extremely small and mobile bacteria were found, but the blood of measles was not toxic to rabbits The authors state that the blood taken from a part of the skin covered with eruptions contains many more bacteridia than the blood taken from the sound part of the skin, and believe, therefore, there is a connection between the eruption and the number of bacteroid elements; in other words, we presume that they mean to affirm a great local development of bacteria in the vessels of the skin.

In the blood of puerperal fever, the authors found (in all infectious diseases) the following changes: deformed red globules, augmentation of leucocytes, fibrillary deposits of fibrine and chains of bacteria, and the same characters were found in the blood of rabbits poisoned with injection of puerperal fever.

The observations of Coze and Feltz on septicæmia are strongly supported by the beautiful experiments of Burdon-Sanderson and Klein, and by the observations of Klebs, of Davaine, and several others. Sanderson and Klein have found bacteria in septicæmic and pyæmic blood, and have also confirmed in another way the curious observations of Coze and Feltz, that by successive inoculations the virus increases in intensity. If a pyæmic fluid is transferred to the peritoneum of a guinea-pig, and is allowed to remain there for a couple of days, and is then introduced into another animal, its toxic power has so increased that it has acquired the most deadly activity. "All such extremely active liquids were crowded with bacteria of a peculiar character, the increased number of which seemed to be in proportion to their toxic properties."

Dr. Sanderson believes that bacteria "afford a *characteristic*, by which we may distinguish the products of infective inflammations from those which are non-infective, and that their number affords an indication of their degree of infectiveness." Dr. Sanderson, however, believes, from actual experiment, that the ordinary bacteria of putrefaction have no toxic action; and he is not prepared, at present, to say that the bacteria of septicæmia are the toxic agents. He regards them as the inhabitants of infective fluids, and as, very probably, carriers of infection.

The work of Klebs, which is based upon observations in Carlsruhe, during the war of 1870-71, is remarkable in various surgical aspects, but in none more than the statements made as to the chief cause of death after wounds. He notices that the differences in the amount and fatality of wound-fevers, and pyæmia and septicæmia, cannot be dependent on the physical condition of the pus, for it is of all kinds of finenesss and coarseness.

A fine microscopic examination has convinced Klebs that the cause of secondary pus formation lies in the presence of "putrefaction fungi" (Faulnisspilzen), which he terms microsporon septicum. He confirms entirely the statements of Recklinghausen and Waldeyer, and, though he does not allude to him, his observations seem confirmatory of Lister's. The method in which it is sought to prove that this parasite is a pus and fever-making cause is two-fold: an anatomical and a physiological. With regard to the first, the microscopic examination showed bacteria, vibriones, and monads in almost all cases of wound secretions. The bacteria are often motionless, rod-like bodies; often joined together, so as to form long jointed fibres; there were, also, numerous microspores, that is, extraordinarily small glistening particles, either free, and then having oscillatory movement, or in groups (zooglœ-form), or in chaplets. These bodies are found in good as well as in bad pus, but they are sometimes wanting in good pus. These parasites are, Klebs thinks, the same as those described by Huctor and Tommasi in the diphtheritis of fresh wounds.

Commencing in the secretion on the outer surface, the parasites attach themselves to the soft parts, and colonize there, and form zooglœa-masses, just as they may be artificially grown on the mesentery of the frog.

The colonies extend themselves on all sides, unless they meet with a cleft, which interposes a chasm, or produces mechanical compression, which destroys them. They destroy the surface in this way, and then they penetrate the lymph and blood-vessels, and get to the inner organs. Sometimes they eat through the wall of a vessel, from outer to inner coat, and, getting into the vessel, cause coagulation.

Of the parasite, many penetrate into soft tissues, which they easily destroy, though the hard bones and tendons resist them. They penetrate into the interstices of the loose connective tissues, either directly or are assisted by those forces which aid in moving the lymph. This latter mode is very important. When the parasite passes into the connecting tissue interspaces, the permanent cells are destroyed by the mechanical pressure, but in the spaces are found the wandered white-cells. The parasites pass into these wandering cells (which have come from the wound surfaces apparently, and contain often hæmatoidin), and then are traced into the lymphatic glands. A general infection of the system occurs very slowly in this manner, and the spread of the parasite-holding cells beyond the lymphatic glands is very difficult to prove. The parasite arrives in the muscles from the connective tissue spaces; then, during the contraction of the muscular fibres, the spaces between them are widened, and neighboring fluids are drawn by aspiration into the spaces. In the succeeding period of muscular relaxation the solid particles in the fluid are not driven out again. Then occurs the well-known interstitial myositis and pus building.

The general infection of the body arises most commonly by the infection of the blood, and the transference to various parts; the little thromboses, which are found behind the valves in the veins, are caused by the adherence of the microspores to the walls; by their colonization and growth there, and by the irritation and pouring out of a fibrino-plastic substance on the walls, a coagulating influence is exerted on the blood, and, perhaps, also the ante-coagulating influence of the walls, pointed out by Brucke, is removed.

These thromboses may remain on, and often form pus. Then the organs suffer; the lungs especially, from the mechanical arrest of numerous solid parts, or sometimes from coagula in the vessels.

Klebs enters at considerable length into the thrombosis and coagulation in the lung vessels in septicæmia, and then passes to the hepatic abscesses, which seldom arise from emboli, but from the distribution of the microspores in the capillary vessels, which are distended, press on the liver cells and destroy them.

Klebs concludes that the opinion which looks upon these parasitic elements as merely unessential and accidental attendants of suppuration and inflammation, must be given up, as complete proof has been obtained that the local mycosis precedes these processes. But, in addition, Drs. Zahn and Tiegel have succeeded in filtering the parasitic masses. The clear fluid caused heavy but transient fever, but never caused local suppurations; the same fluid, containing the parasites, caused extraordinary wide-spread suppurations. Zahn's experiments have, however, been doubted.

Finally, in the splenic apoplexy, or carbuncular diseases, (Milzbrand) of sheep and cattle, in which bacteria were discovered by Brauell, Davaine, and others, it has been asserted by Dr. V. Grimm, of St. Petersburg, that no bacteria were found in the blood during life. But this has been contradicted in a note by Dr. Semmer, of Dorpat, who, not only from his own observations, but from those of Unterberger, in Dorpat, and Nayorski, in St. Petersburg, entirely confirms Brauell's statement of the constancy of the occurrence of these bacteridia in the blood of carbuncular disease.

All of the above noted affections belong to the strongly marked class of infectious diseases; but some observations have also been made on fatal cases, which are allied to these infectious disorders.

NON-SPECIFIC DISEASES ATTRIBUTED TO BACTERIA.

*Mycosis endocardii.*

Under this term, Professor Winge, of Christiana, described, in 1869, a case of ulcerous endocarditis, in which there were numerous fungoid filaments on the several valves, and in the little embolic masses which were found in the heart. The vegetations were like fibrinous threads, but, under an immersion-lens, their characters came out clearly; there were partitions, and the threads were branched. There was detritus and granular heaps, very similar to bacteria rods, while the threads were like leptothrix-mycelium. The case came on after the man had opened a suppurating corn, with repeated shiverings, followed by severe headaches, diarrhœa, and typhoid-like symptoms.

Another case of a similar kind is now recorded by Professor Heiberg, of Christiana. It was the case of a puerperal woman, who, on the tenth day, had shivering and vomiting, followed by swellings in some joints, and vesicles on the skin of the extremities. She died in about five weeks. On *post-mortem* examination, there was ulcerous endocarditis, and thrombosis of the mitral valve, with fungoid growth. There were metastatic abscesses, metro-lympho thrombosis, and lung œdema, and hyperæmia. On a microscopic examination of the cardiac valves, and of the coagulæ adhering to the chordæ tendinæ, there were numerous fine granules, and many rod-like bacteria—similar bodies and leptothrix chains. There was no fibrine, but many white corpuscles in the thrombus masses. The chains and links entirely agreed with those observed in Winge's case. The author gives reasons why in both cases, the plant could not have been a *post-mortem* appearance, but that they were developed *intra vitam.* Detached portions of the vegetation produced emboli. The plant is referred to the schizomycetes (of De Bary), and not to the true fungi.

A sample was transmitted to Virchow, who considers the granules decidedly of parasitic nature, and to be vibrional.

The case is very similar to two described by Virchow, of puerperal ulcerous endocarditis, and in which, also, there were peculiar granules in hyaline connecting masses, and which Virchow identified as parasitic.

DIPHTHERITIC NEPHRITIS CAUSED BY SCHIZOMYCETES.

Dr. Letzwich describes a case of diphtheritis of both tonsils, and swellings in the neck, in a child two and a half years old, which was followed by suppression of the urine, and death. On examination, the venal tubules (straight and contorted), and the malpighian corpuscles, were found crowded with the spores of a fungus; where the fungoid masses were most numerous, the epithelium had disappeared, and the fungi lay close to the basement membrane. The canals were greatly enlarged by their pressure, and the vessels compressed. If the epithelium still existed, it was crowded with spores, and often enlarged to double its natural size. No fungi were found in the spleen or liver, but they were present in the arteries of the kidneys. The cessation of the urinary secretion was purely mechanical. Although the writer uses the term fungus (pilus), his figures only show small round cells, like spores certainly, but which might possibly be bacteroid.

### UTERINE DIPHTHERITIS AND BACTERIA.

Waldeyer describes four cases of puerperal fever, with diphtheritic exudation on the internal surface of the uterus; this layer, and the contents of the lymphatics, the co-existent peritoneal, pleuritic, and pericardiac exudations, were found to contain masses of bacteria between the pus corpuscles and the mortified tissues, and bacteria were found even in the pus corpuscles. The form was the "Kugel-bacteria," of Cohn (already noticed.) While Waldeyer does not lay an extreme weight on these appearances, he considers they cannot be indifferent, and refers to the nephritis diphtheritic of Klebs and Recklinghausen. His observation on the presence of bacteria within pus cells is confirmatory of Beachamp's and Estor's observations, and of Bastian's remark on the presence of bacteria in living cells.

### BACTERIA IN THE VESSELS OF THE BRAIN.

In a case of rheumatic fever, which died with an excessively high temperature, Bastian found outside and in the central vessels (surface of convolutions), a large number of actively moving particles. many of these were distinct and large bacteria, made up of "two almost cellular segments." Bastian says these bacteria must have existed in the blood during life, or have been produced in the vessels after death, and before the skull was opened. He inclines to the latter opinion, and states that it influenced him in his views of spontaneous generation. But there are now so many cases of bacteria in the blood during life, that it seems much more probable there was some "foyer" which furnished those found in this rheumatic case.

